# Introduction to effective mentorship for early-career research scientists

**DOI:** 10.1186/s12919-021-00212-9

**Published:** 2021-06-22

**Authors:** Kelly A. Diggs-Andrews, D. C. Ghislaine Mayer, Blake Riggs

**Affiliations:** 1 Diggs-Andrews Consulting LLC, Ashburn, VA USA; 2 Manhattan College, Department of Biology, Riverdale, NY USA; 3 Biology Department, San Francisco State University, San Francisco, CA USA

**Keywords:** Mentoring, STEM, Underrepresented scientists, Faculty development

## Abstract

Diversifying the scientific workforce remains a national priority due to the continued lack of representation from underrepresented individuals in STEM fields. Quality mentoring has been identified as a stimulus to enhance not only research success, but also recruitment and retention of underrepresented groups pursuing STEM careers. Utilizing the *Entering Mentoring* training curriculum framework, this report provides a brief synopsis and key takeaways from the 2019 NIH-ASCB Accomplishing Career Transition (ACT) workshop, “Introduction to Effective Mentorship for Scientists” for 30 senior postdoctoral and early-career faculty researchers from historically underrepresented racial and ethnicity backgrounds. In addition, effective strategies and best practices to enhance STEM mentoring for early-career researchers are provided, which have practical applications for diverse mentoring relationships across disciplines, career stages, and mentee types.

## Background

Scientific investigators are always seeking more effective strategies to build diverse research teams and increase research productivity [1]. While there have been considerable efforts over the past 40 years to promote diversity in science, technology, engineering, and mathematics (STEM), unequal representation of individuals from Black or African American, Latinx or Hispanic backgrounds, and those indigenous to the United States and its surrounding territories (including American Indians or Alaska Natives, Native Hawaiians, and other Pacific Islanders) still exists in many STEM fields [2]. As illustrated in recent PEW reports, although African-American and Latinx workers comprise 11 and 6% of the overall U.S. workforce, respectively, these groups represent only 9 and 7% of STEM workers. Moreover, this underrepresentation in STEM jobs appears to be further exacerbated among employed adults with bachelor’s, professional, or doctoral degrees [3]. This is especially true in biomedical research, which displays significant underrepresentation at the faculty and Principle Investigator level.

Recent reports have suggested that quality mentoring can positively impact both lab dynamics and research success. Specifically, positive mentoring experiences have been linked to increased productivity, career satisfaction, and research success of trainees [4–11]. For individuals currently underrepresented in STEM areas, strong mentorship has been shown to increase recruitment, retention, and persistence in science careers [12–16]. Recruitment efforts targeting underrepresented minority students by colleges and universities often include workshops and campus tours for middle and high school students. Additionally, some institutions host specific minority recruitment weekends and operate minority recruitment programs supported by their Offices of Admissions. At the undergraduate and graduate level, multiple interventions have aimed to increase retention of underrepresented minority students in STEM careers. Most notably, minority-focused mentoring and training programs, including the Meyerhoff Scholarship Program, MARC/U-STAR (NIH–NIGMS Maximizing Access to Research Careers/Undergraduate Student Training in Academic Research), LA-STEM (Louisiana Science, Technology, Engineering & Mathematics), Ronald E. McNair Postbaccalaureate Achievement Program, and SACNAS/Synapse (Society for Advancement of Chicanos and Native Americans in Science/Supporting Young Native Americans to Pursue Science Education), have demonstrated solid track records of retaining minority students in STEM fields [17].

While the importance of mentoring is widely recognized, few structured training interventions exist for (1) novice mentors to acquire evidence-based skills or (2) experienced mentors to develop new approaches and innovative mentoring practices [18–20]. Further, many mentors are often unaware of available resources or training opportunities due to limited or restricted dissemination of mentoring best practices. As such, these mentors rely primarily on prior knowledge and observational experiences to inform their mentoring principles, and do not receive the benefit of exposure to effective, verified solutions to enhance their mentoring practices [20–22]. Therefore, in an effort to strengthen and diversify the scientific enterprise, the American Society for Cell Biology (ASCB) sponsored a four-day professional development workshop, Accomplishing Career Transitions (ACT) on June 24–27, 2019 in Chapel Hill, NC, for 30 early-career faculty and postdoctoral researchers, primarily comprising of members from underrepresented groups (i.e. persons who identify as Black or African American, Hispanics or Latinos, American Indians or Alaska Natives, Native Hawaiians, and other Pacific Islanders). This report discusses the two-hour evidence-based interactive mentor training seminar, led by Master Facilitator Dr. Kelly Diggs-Andrews, utilizing the NIH-National Research Mentoring Network (NRMN) *Entering Mentoring* [19] mentor training curriculum. This training workshop has been previously demonstrated to increase skill gains and support behavioral changes across multiple mentoring domains needed for successful mentoring relationships, including maintaining effective communication, aligning expectations with trainees, and addressing issues of equity and inclusion [18–20]. Therefore, the primary goals of the workshop were to build awareness of evidence and available resources to promote effective mentoring practices for mentors of diverse undergraduate and graduate trainees. Moreover, the training provided numerous best practices and guided activities to help participants identify their mentoring core strengths and initiate development of their mentoring philosophy statement. Additionally, identification of peer and near-peer mentoring allies within their cohort was encouraged to continue conversations and learning as they tackled common mentoring challenges. Specific learning objectives of the workshop were as follows:

1. Describing the core elements of mentoring, including maintaining effective communication, establishing expectations, and addressing equity and inclusion.

2. Clearly articulating expectations for the mentoring relationship.

3. Practicing effective strategies for improving communication.

4. Recognizing the impact of biases, prejudices, privilege, and power on the mentor-mentee relationship and acquiring skills to manage them.

5. Identifying the roles mentors play in the overall professional development of their mentees and work-life integration using Stewart Friedman’s Total Leadership model [23].

In this report, key insights and discussion that emerged from this mentoring workshop are highlighted to foster and cultivate effective mentor-mentee relationships for early-career STEM researchers. Moreover, these strategies and mentoring principles can be practically applied across various disciplines and career stages of mentoring relationships to enhance mentor engagement, mentee success, and support scientific workforce diversity.

### Best practices in STEM mentoring

#### Addressing culture and diversity: challenges and opportunities

Effective mentorship can fulfill several critical purposes, including career advancement, role modeling, and psychosocial functions [24]. In particular, in STEM professions, mentors support integration into professional and lab cultures by helping their mentees acquire professional identities and networks [25]. Evidence has shown that mentoring is critically important for the successful outcome of underrepresented individuals and women in STEM fields [2, 4, 5, 13–19, 26]. Effective mentoring can combat the ongoing pressures that underrepresented individuals face due to implicit bias by colleagues and supervisors, macro- and microaggressions, stereotype threats felt by the mentee in toxic biomedical training environments, and social isolation in part due to underrepresentation within the field [2, 27]. Many reports have noted challenges with accessibility and barriers to STEM careers for underrepresented students [28]. Moreover, gender differences in mentorship and sponsorship have been suggested as contributing factors to the perpetuation of male-favored advantages in STEM disciplines [29]. This exclusion is often compounded by a lack of self-confidence, self-efficacy, and imposter syndrome [30]. However, despite positive intentions, many mentors feel ill-equipped to tackle these issues [31] due to the absence of proper mentor training and interaction with diverse populations. Thus, bias, diversity, and professional development trainings have been recommended as a time- and cost-effective strategy to enhance the effectiveness of STEM research mentors [26, 32].

Additionally, STEM mentees have reported a lack of sensitivity to the importance of race, gender, and class in the mentoring relationship [33]. Often, the historically advantaged population do not recognize the unique experiences of those who are overlooked because of institutionalized discrimination and unconscious bias [34]. Moreover, most current applications of mentoring principles further advantage well-represented individuals in many institutions, putting women and underrepresented minorities at a greater disadvantage in most fields and in leadership positions [35, 36]. Therefore, in fostering mentoring relationships with underrepresented trainees, it is critical to recognize the importance of engaging in critical conversations about culture and diversity and acknowledging their unique cultural and life experiences, barriers, and skills [37, 38].

#### Promoting multiple mentors and mentoring networks

The benefits of mentoring in the growth and advancement of scholars and scientists are widely documented [39, 40]. For effective mentorship, relationships should be multi-dimensional, while addressing numerous and disparate objectives. Mentoring is a socialization process that is highly reliant on the development of collaborative, social, and mentoring networks. However, underrepresented minorities have often reported feelings of exclusion from these socialization experiences [2, 41]. In addition to these roles, a mentor must support, inspire, challenge, affirm, coach, counsel, protect, and provide constructive feedback. These varied roles cannot be fulfilled within any single mentor-mentee relationship [42]. Thus, mentoring networks composed of multiple mentors should be encouraged to ensure adequate availability and support for each mentee [26].

An ideal mentoring network should consist of near-peer, peer, and established professional mentors. Near-peer mentoring is especially beneficial for many trainees who experience challenges when transitioning into higher education, i.e. acclimatization to the new environment and academic expectations. For undergraduate and graduate students, near-peer mentoring is an established relationship between students, in which one student is 1–4 years senior to the other student, on similar academic tracts, and within the same institution and/or laboratory environment [43]. Due to the proximity in age, similarities in academic and social circles, and overlap in courses, near-peer mentors provide a unique and unparalleled support structure for their mentees. For example, in a near-peer mentoring model comprised of STEM post-baccalaureate and undergraduate students in STEM research and education at seven institutions, it was shown to promote career advancement and psychosocial support associated with acquisition of professional behaviors [44]. Additionally, comparable near-peer mentoring programs in medical institutions have been shown to increase student retention and enable academic success [45].

Similarly, peer-mentoring, also called horizontal mentoring, is a valuable type of mentorship between students or student groups who are new to a given field, subject matter, or campus. Peer-mentoring has been shown to accelerate academic and professional development of individuals by providing reciprocal support and collaboration [18]. Moreover, peer-mentoring has been shown to be highly beneficial to both undergraduate and graduate students, especially for those from underrepresented backgrounds, by enabling trainees to overcome feelings of isolation [46, 47]. Junior faculty members also benefit from peer-mentoring. In programs, such as the Vanderbilt University Peer Mentoring Program [48], cohorts reported significant skill gains in professional development and scholarship, in part due to increased interconnectivity with other faculty members. Similarly, a very popular and useful tool for new investigators NewPI Slack, a global peer-mentoring platform, was created to provide community for Assistant Professors [49].

Further, mentees should be encouraged and supported in creating a developmental network of established professional mentors, beyond their primary mentors. This will usually involve informal mentoring pairs, where established professionals work to understand the mentee’s overall professional, research, or careers goals in order to support the mentee in achieving a specific goal(s). These mentors are both accessible and committed to helping the mentee, while also providing a different perspective from the primary mentor. Developmental networks have also been shown to be beneficial for mentors as these mentoring networks can provide support for both the mentee and primary mentor if difficulties arise [50]. Therefore, for mentors, identification of mentoring allies both within their home institutions and at external institutions is a strategy to create a community of practice of committed mentors that are continuously learning new approaches to enhance their mentoring practices, thus receiving/providing advice and support, sharing resources, and discussing mentoring challenges and solutions.

An important role of mentors is to foster the professional development of their mentees. In STEM fields, mentees often train for a specified duration with their primary mentors, followed by employment or additional training. To support discussions regarding professional development, the Individual Development Plan (IDP) is a highly recommended, yet often underutilized tool. First developed for postdoctoral fellows in 2002 [51], IDPs have been adapted to help mentees across career stages and disciplines to delineate their career goals and outline plans for achieving those goals. Moreover, IDPs have been shown to be most effective and useful when incorporated in the mentoring relationship [52]. As a result, both the National Institute of General Medical Sciences (NIGMS) and the National Institutes of Health (NIH) encourage development and recommend annual review of IDPs for graduate students and postdoctoral fellows of federally supported investigators [53, 54].

In addition, a significant benefit of having mentors is access to resources and networking opportunities. Networking entails establishing enduring contacts that can boost one’s STEM career. While networking is often considered within the framework of a job search, it is also critical for sustained success in STEM fields for both mentors and mentees. Specifically, networking opportunities can boost visibility of mentees while increasing contact with established and potential collaborators for mentors. Networking can also provide a source of external career advisors for mentees.

### Mentoring tools

#### Aligning expectations using mentoring compacts

In a successful mentoring relationship, mentors and mentees should establish regular meetings to get to know each other, which can be conducted either formally or informally, and clearly express their expectations of the mentoring relationship. This can be done by documenting short- (1 year), mid- (2–5 years), and long- (> 5 years) term professional goals as part of a written career development plan or mentoring compact [55, 56]. Mentoring compacts and comparable documents give mentees an opportunity to align their expectations with their mentors, provide clear direction for the research project, and ensure that mentees obtain essential skills to achieve their goals. When using these tools, it is helpful to adopt the SMART (Specific Measurable Achievable Relevant Timely) goal framework [57], which includes asking targeted questions about the specific task or goal, its significance, and the necessary resources, effort, and commitment required to achieve successful implementation. These goals should be reviewed with prospective mentors to determine suitability prior to a potential mentor-mentee match. Mentees should also consider the mentor’s experience, availability, training opportunities, resources, and commitment to help them reach their goals. Subsequently, discussions regarding expectations for the mentoring relationship can be supported by outlining the roles of both the mentor and mentee, specifically what will be accomplished, how it will be accomplished, by whom, and when. Outlining expectations early on and revisiting them frequently can limit challenges associated with miscommunication or misunderstanding. In a research context, these efforts can further clarify the scope of the mentee’s project and identify opportunities for collaboration and authorship, thus avoiding significant overlap between projects and potential competition with mentors and other lab members. An additional benefit of aligning expectations is fostering mutual trust and respect. Moreover, mentors are more likely to honor their commitment when expectations are clearly delineated early in the mentoring relationship.

#### Developing a mentoring philosophy or action plan

To aid in clarifying and developing your mentoring approach and style, there are several tools available, including, but not limited to, a mentor philosophy or mentoring action plan [58]. By developing a mentoring philosophy and thinking intentionally about the goals of each mentoring relationship, mentors can strategically direct their mentoring efforts and determine how to support their trainees if and when issues arise. In addition, it allows mentors to define the type of mentor they wish to be, which is often overlooked and shaped based on prior mentoring experiences [38, 59, 60]. When initiating a new mentoring relationship, both the mentor and mentee must first assess the goals of the mentoring relationship and how the mentor can best support the mentee. Specifically, a mentoring philosophy should explain the mentor’s approach in guiding the new partnership. Preferably, the mentoring philosophy should be individually tailored to each mentor-mentee relationship, including identifying the mentor’s and mentee’s goals (individual and collaborative) and motivation, developing engagement norms for the relationship, and discussing assessment tools to evaluate the mentee’s learning and the effectiveness of the relationship [61–63]. Ideally, the mentee should lead the discussion regarding the overall goals and expectations for the mentoring relationship, with guidance from the mentor. However, any unifying principles, professional norms, and guidelines that would apply to all mentees should be addressed by the mentor. Once 3–5 mutually agreed goals are established, a plan of action and a timeline to achieve these goals should be set [61–63]. These can be learning activities, specific steps towards achieving each goals, or sub goals, with the timeline establishing a reciprocal accountability structure and enabling the mentee to visualize the path to achieving these goals. Taken together, this initial effort and discussion at the start of the mentoring relationship can greatly shape the path forward and provides a foundation for an effective mentoring relationship.

#### Addressing difficulties in the mentoring relationship

When faced with challenges in a mentoring relationship, it is important for the mentor to first recognize when an issue arises and identify its source. Due to the inherent power dynamic in mentoring relationships, it can be difficult for mentees to see problems and subsequently approach their mentors to address these issues. However, the mentee shares responsibility to nurture and proactively engage in the mentoring relationship by managing or “mentoring” up [64]. As mutually beneficial partners, mentoring up requires mentees to increase awareness of their needs and contributions in the mentoring partnership. It also encourages mentees to take agency in navigating the mentoring relationship by clearly articulating their learning and career objectives to their mentors, as well as assuming responsibility and accountability for their actions and behaviors. Because a foundational knowledge of mentoring and effective mentoring practices is needed for both mentors and mentees, evidence-based workshops and complementary training curricula, including *Entering Research* and *Mentoring Up*, have been developed to support mentees as they learn to mentor up at all career stages [65–67].

When issues are present, it is imperative that the mentor and mentee engage in a difficult conversation to keep the mentoring relationship on track. There are several strategies to approach these conversations [68–70]. First, these conversations should occur in an unintimidating, safe space. Ideally, the place should not perpetuate the power differential in the mentoring relationship (i.e. mentor’s office). If possible, mentors should conduct an environmental scan of their offices and meeting spaces to support open and inclusive dialogue with their trainees. Alternatively, mentors can engage in these conversations in spaces that are emotionally neutral, such as a public space (park, café, conference room, shared break area, etc.) or in a setting or low-risk activity that a mentor would normally engage with the mentee. Second, these conversations should be completely confidential. This reinforces the level of trust in the relationship and will set a tone for honesty and openness. Third, the mentor should practice active listening strategies to focus completely on the conversation without distractions [71, 72]. Distractions—email alerts, text messages, or calls—can lessen the seriousness of the conversation and may devalue the topic being discussed. To demonstrate engagement, the mentor should ask open-ended questions to allow for different personal perspectives to be shared and feelings to be expressed. While this may be difficult for faculty as many relish the opportunities to share advice, this can shut down a dialogue and not allow for an open exchange. Mentors are also encouraged to share personal stories, experiences, and challenges, as appropriate, to humanize, display empathy, and provide inspiration. Finally, it is key to recognize that these issues may not be solved in one conversation. Ongoing, regular discussions can be beneficial to take small steps towards solving a problem or mitigating new issues. However, for significant issues that cannot be resolved, requiring the mentoring relationship to end, these discussions enable both the mentor and mentee to fully process the discussion, generate ideas and solutions to solve the issues, and hopefully end on good terms.

## Conclusion

This report describes best practices and resources to support effective mentorship of STEM researchers. These evidence-based strategies, such as developing mentoring networks and addressing culture and diversity, have been shown to positively impact mentoring relationships across career stages, disciplines, and mentee types. Therefore, by leveraging available mentoring resources (i.e. mentoring compacts, mentoring philosophy/action plans, etc.) when initiating new mentoring partnerships, early-career mentors are enabled to implement specific actions to enhance their mentoring practices and principles with diverse mentees, thus contributing to a skilled, diverse, and sustainable scientific workforce.

## Data Availability

Not applicable.

## Abbreviations

ACT: Accomplishing Career Transitions
ASCB: The American Society for Cell Biology
NIGMS: National Institute of General Medical Sciences.
NIH: National Institutes of Health
NRMN: National Research Mentoring Network
STEM: Science, Technology, Engineering, and Mathematics
